# A TRIZ Approach for Designing a Smart Lighting and Control System for Classrooms Based on Counter Application with Dual PIR Sensors

**DOI:** 10.3390/s24041177

**Published:** 2024-02-10

**Authors:** Peng Lean Chong, Daniel Ismail, Poh Kiat Ng, Feng Yuan Kong, Mohammed Reyasudin Basir Khan, Sargunam Thirugnanam

**Affiliations:** 1 School of Engineering and Computing, MILA University, No 1, MIU Boulevard, Putra Nilai, Nilai 71800, Malaysia; sargunam.thirugnanam@mila.edu.my; 2 Faculty of IT and Technology, International University of Applied Sciences Bad Honnef, 53604 Bad Honnef, Germany; daniel.ismail@iu-study.org; 3 Faculty of Engineering and Technology, Multimedia University, Jalan Ayer Keroh Lama, Melaka 75450, Malaysia; fykong@mmu.edu.my; 4 Universiti Tun Abdul Razak (UNIRAZAK), Wisma UNIRAZAK, 195A, Jln Tun Razak, Hampshire Park, Kuala Lumpur 50450, Malaysia; reyasudin@unirazak.edu.my

**Keywords:** smart classroom, energy saving, human presence detection, passive infrared sensors

## Abstract

Electrical energy is often wasted through human negligence when people do not switch off electrical appliances such as lighting after leaving a place. Such a scenario often happens in a classroom when the last person leaves the class and forgets to switch off the electrical appliances. Such wastage may not be able to be afforded by schools that are limited financially. Therefore, this research proposed a simple and cost-effective system that can analyze whether there is or is not a human presence in the classroom by applying a counter to count the total number of people entering and leaving the classroom based on the sensing signals of a set of dual PIR sensors only and then correlating this to automatically turn on or off the electrical appliances mentioned. The total number of people identified in the classroom is also displayed on an LCD screen. A TRIZ approach is used to support the ideation of the system. The system can switch on several electrical output loads simultaneously when the presence of people is detected and switch them off when there are no people in the classroom. The proposed system can be expanded to be used in homes, offices, and buildings to prevent the high cost of electricity consumption caused by the negligence of people. This enables smarter control of electricity consumption.

## 1. Introduction

High consumption of electrical energy leading to high costs is often reported in various countries. In 2010, it was found that the cost of lighting accounts for about 10% of total electricity consumption in buildings and homes in the United States [1,2]. In addition, the education sector alone consumed 10% of total electrical energy consumption in the United States in the year 2012, thus, making it the third largest energy-consuming sector in the country [3]. Electricity was mostly consumed to operate primary schools, middle schools, high schools, colleges, and universities. Hence, energy conservation is considered a fundamental aspect of facility management to enable cost-efficient buildings in educational institutions. Therefore, the solution revolving lower energy consumption is often set as a target for cost reduction initiatives.

Often, high electricity consumption in educational institutions is caused by electricity wastage which can be prevented. Such electricity wastage normally occurs when electrical devices, such as lighting, are turned on, even when no one is present in the classroom because the last student leaving the class forgot to switch off all the electrical loads [4]. Such a scenario is widely seen in schools and universities where the classrooms or lecture halls have switched on lighting, and no one is in the venue [4]. This condition serves no beneficial purpose and contributes to the wastage of electricity due to human negligence. Previous studies have shown that occupant presence and behavior in buildings have a major impact on energy consumption for lighting [5]. Thus, careless user behavior can influence the energy efficiency of a building by one-third, which is a significant contributing factor [6]. Therefore, autonomous energy-saving initiatives must be implemented to reduce the careless behavior of students that leads to the wastage of electricity in the classroom environment.

### 1.1. Research Contribution

This research contributes to providing an autonomous energy-saving method for turning on or off appliances by counting the number of individuals present to overcome the careless behavior of students that forget to switch off the lighting before leaving the classroom which leads to the wastage of electricity. It also resolves the problem of the traditional existing off-the-shelf system for classroom application by using a PIR sensor that works on direct human motion detection and requires continuous body movements such as clapping or moving hands to enable continuous stimuli to the PIR sensor. This study designed a cost-effective smart lighting and control system that showcases a novel method to utilize dual PIR sensors paired with counters to autonomously identify the number of people in a classroom and decide to switch on or off the electrical appliances based on the total number of human presences detected. A dual PIR sensor strategy is used to detect the total number of persons entering and exiting the classroom, and the acquired data is input into the system for counting. The proposed solution will contribute to energy savings, lower electrical costs, and intelligent home lighting management for long-term use.

### 1.2. Paper Organization

The entire article is organized as follows: Section 2 explains the literature review of the past research and identification of the research gap, Section 3 will emphasize the materials and methods covering the TRIZ conceptualization methodology used and explains in detail about the fabrication of the designed prototype, Section 4 discusses the result, testing and validation of the developed system, Section 5 discusses the limitation of the system and its prospects and Section 6 concludes the article.

## 2. Literature Review

In the past 30 years, most of the present off-the-shelf systems have utilized passive infra sensors (PIR sensors) to sense human presence to automate the triggering of electrical appliances in a venue [7]. Numerous research has revolved around the application of PIR sensors with a trigger circuit for switching electrical appliances [8,9,10]. Examples are shown by Mat and Suriza who presented a detailed exploration of the technical aspects of a green technology system designed for classroom application [11]. The researchers proposed an autonomous lighting system that can minimize electricity usage by using sensors to detect the presence of humans and control the intensity of the lamp. The three sensors used by this proposed system are a light sensor, a passive infrared (PIR), and an infrared (IR) sensor which can all function independently but can also occasionally operate simultaneously to measure and analyze light brightness in relation to environmental exposure. However, the prototype still depends on continuous human motion to trigger the output loads and it will have the risk of the system switching off the electrical loads if no human motion is detected for a long duration. With no continuous movement to trigger the PIR sensor even though there are still people in the room, this can lead to inconveniences where movements are needed from time to time to continue the operation of the electrical loads.

Moreover, as PIR sensors suffer poor precision if used to detect human motion directly, many alternative methods are being explored to accurately locate human presence to correlate it to electrical loads triggering. Lai et al. [12] proposed the usage of two pyroelectric infrared sensors to be embedded into a non-terminal indoor localization system mounted on the ceiling to increase the precision in estimating the location of a target inside a room. In addition, the detection of human presence using a fuzzy logic approach is presented by Chowdhury and Tripathy in [13] where the researchers focused on the utilization of a fuzzy approach to detect human presence in surveillance videos. The authors delve into the application of fuzzy logic, a computational approach that deals with approximate reasoning, to address the complexities and uncertainties inherent to human presence detection. The study explores the technical aspects of implementing fuzzy logic algorithms to analyze video data and identify human presence. In addition, Joshi et al. presented a study in [14] that focused on human detection using explicit skin color space thresholding and minor motion detection techniques to accurately detect human presence in visual data. By combining explicit skin color space thresholding with minor motion detection, the authors aim to enhance the robustness and accuracy of human detection systems, particularly in scenarios where traditional methods may be susceptible to environmental variations or noise. Furthermore, Asakipaam et al. present a study that focuses on the development of a smart energy conservation system for study rooms, which includes the regulation of lighting based on the presence of humans using relay control and mobility [15]. While the study specifically mentions study rooms, the principles, and technologies employed in this system are highly relevant to the automation of lighting in classrooms based on human presence detection. Further studies on smart lighting control systems are explored in [4] where Suresh et al. present an Arduino-based automatic lighting and control system connected to relay control to detect human presence in a classroom condition which is divided into several grid sections. The prototype is also coupled with an Android mobile app and Bluetooth control to enable remote command execution to control the lighting with voice command.

With the technology proposed by previous research stated above in enabling a smarter lighting and control system for classrooms, only a few of them are focused on finding alternative methods to use existing PIR sensors in a smarter way to enable higher precision and accuracy for detecting human presence to control electrical appliances. Most of the research diverts away from optimizing PIR sensors application and focuses on other more advanced technologies. However, as the utilization of PIR sensors are still the norm in the industry for human presence detection due to its cheap cost, ease of usage, and wide availability in the market for more than 30 years [7], it is still the preferable choice of electronic component being incorporated into solutions for various industries, particularly in smart classroom control systems. Besides that, the problem of the traditional existing off-the-shelf system using PIR sensors for classroom application, which works via direct human motion detection and needs continuous body movements such as clapping or hand movements to enable continuous stimuli to the PIR sensor, also should be resolved. Therefore, this research aims to fill this research gap by designing a cost-effective smart lighting and control system that showcases a novel method to utilize dual PIR sensors paired with counters to identify the number of people in a classroom autonomously and decide to switch on or off the electrical appliances based on the total number of human presences detected. A pair of PIR sensors are installed at the entrance and exit door to sense the total amount of people entering and the total amount of people leaving the classroom and then the data is fed to the system for counting. This solution offers an intelligent method to switch on or off appliances by counting the number of humans present and does not depend on sensing continuous human movements as is the case in traditional existing systems. As a summary, a comparison table is portrayed in Table 1 to compare the various existing approaches addressed above with the proposed method here to highlight the novelty of the solution proposed.

Hence, to enable a systematic methodology to foster creativity and innovation in the design of the prototype, the “Theory of Inventive Problem Solving”, more widely named as TRIZ problem-solving tool is used for the ideation of the cost-effective smart home appliance activation system. The theory of inventive problem solving (TRIZ) serves as a framework to facilitate the ideation process. TRIZ, an internationally recognized creative methodology, was formulated over the course of several decades, from 1946 to 1985, by the engineer and scientist Genrich S. Altshuller, in collaboration with his associates within the USSR. This innovative approach has demonstrated its effectiveness across diverse domains, encompassing fields such as architecture, automotive engineering, financial institutions, construction, and the design of energy-efficient products [16,17,18,19,20]. This study elects to leverage TRIZ for concept selection due to its adaptability and established efficacy in a variety of domains. The TRIZ methodology has been referenced in numerous scholarly works [21,22,23,24] and involves the following procedural steps:

1. Articulation of the engineering contradiction.
2. Determination of system parameters pertinent to the engineering contradiction.
3. Overlapping of system parameters within the TRIZ contradiction matrix.
4. Adoption of an inventive principle derived from the intersection of system parameters.
5. Generation of a solution based on the chosen inventive principle.

The formulation of the engineering contradiction can be accomplished through the application of the if-then-but framework in TRIZ. Within this framework, the “if” statement denotes a manipulative variable, the “then” statement signifies a responding variable that exhibits positive changes due to the manipulative variable, and the “but” statement represents another responding variable that experiences adverse changes because of the initial responding variable. In the context of this study, the if-then-but contradiction statement is constructed based on the contradiction observed concerning the demand for energy services and the concurrent utilization of multiple electrical appliances.

Every responding variable within the contradiction statement is associated with specific system parameters within the TRIZ framework. TRIZ encompasses 40 distinct system parameters (as delineated in Table 2), with each parameter denoting a factor that delineates a system, establishes, and confines a system’s performance, or characterizes the attributes of a system [25].

Once the appropriate system parameters have been selected for association with the contradiction statement, they serve as indicators that are cross-referenced within the contradiction matrix. The contradiction matrix is a 40 × 40 matrix comprising the system parameters, and it identifies one or more of the 40 inventive principles designed to address a recognized contradiction. Resolving engineering contradictions is most effective when coupled with the application of these inventive principles, as they represent general guidelines for solving technical challenges.

The objective of employing the TRIZ approach in this study is to stimulate innovative thinking aimed at resolving the engineering contradiction to find the optimum approach to enable a smart control system of lighting in the classroom with the usage of existing cheap PIR sensors while enabling no dependency on constant movement of users in the venue to facilitate human presence detection. This contradiction is formulated concerning the primary issues of enabling a smart light control system at a cheap cost for implementation in educational institutions to resolve the problem of human negligence in not switching off the lighting before exiting the classroom. The overarching aim of this approach is to streamline the decision-making process in the context of concept selection.

## 3. Materials and Methods

This section shall explain the ideation process using the TRIZ for the conceptualization of the proposed solution to resolve the contradicting factors and later progress to elaborate on the design of the smart lighting and control system for the classroom.

### 3.1. The Theory of Inventive Problem Solving (TRIZ) Applied

The engineering contradiction (EC), system parameter (SP), innovative principle (IV), and cause-and-effect chain analysis (CEC) were the TRIZ tools employed in this investigation. Initially, the CEC analysis aids in determining the accurate cause or causes of the issue. An ineffective response will result from identifying the incorrect cause. Essentially, CEC analysis is like the “5 Whys” method, which involves asking “why” repeatedly to uncover the causes of the issue as you go from high-level to low-level causes. A few factors have made the CEC analysis one of the more widely used tools: its easy-to-understand principles, its versatility in handling a wide range of complex problems, its capacity to analyze down to the atomic level when needed, and the ease with which its results can be disseminated. The CEC analysis on this research topic starts with identifying the problem to find the root cause of the issue and to distinguish the EC as below.

**Figure 1:**
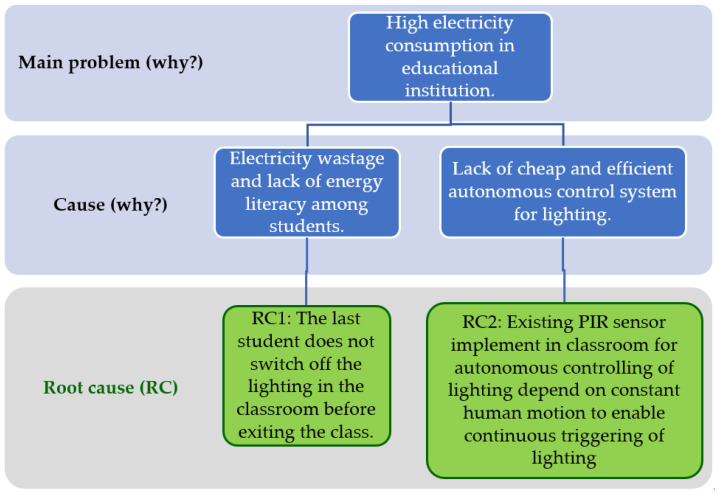
Cause-and-effect chain (CEC) analysis.

The problem of increasing electricity consumption has been reported more often in educational institutions in recent years [26,27]. Hence, the initiative to minimize energy wastage and promote energy literacy among students is commonly implemented in various educational institutions nowadays [28,29]. However, most modern-day classrooms are still facing the dilemma of electricity wastage due to negligence from students not switching off the lighting before exiting the venue [4]. In addition, the lack of a cost-effective autonomous lighting control system applicable for large-scale implementation to resolve this issue among educational institutions is also another problem. Such a system needs to be cheap and affordable due to the financial limitations faced by most of the educational institutions; especially the rural schools which often face budget constraints [30]. However, many of those autonomous light control systems are made with other more expensive technology [4,13,14,15] making them unaffordable for rural schools or they are based on human presence detection which are still using conventional PIR sensors which is dependent on constant human motion to enable continuous triggering of lighting [11,12,31]. Thus, constant movement of teachers and students in the classroom is needed such as clapping the hand whenever the light is off to enable triggering of the light to be switched back on. Figure 1 shows the CEC analysis performed for the main problem.

Hence, the two root causes for the main problem are as follows: (RC1) students do not switch off the lighting before exiting the classroom; (RC2) the existing light control system used is not user-friendly as it is highly dependable on constant human movement detection using conventional PIR sensors. By using the if-then-but algorithm of TRIZ, two relevant EC statements can be formulated:

*EC1 (based on RC1): If the root cause of high electricity consumption in the educational institution is caused by electricity wastage, then such electricity wastage caused by the students who do not switch off the lighting before exiting the classroom should be avoided, but this requires an intelligent autonomous system to control the switching of the lighting based on human presence detection to avoid dependency on human manual control.*

*EC2 (based on RC2): If the existing autonomous lighting control system is not feasible or cost-effective for large-scale implementation in the school, then such a system must be modified from the existing system to avoid huge changes incurring additional costs, but the current cheap system is mostly designed with conventional PIR sensors which depend on constant human motion to enable continuous triggering of lighting.*

After identification of the CEC and EC, we proceed next to find out the SP and IV. As the 40 TRIZ system parameters are connected to the “then” and “but” sections of the ECs, mostly all general system characteristics can be fixed and established by the 39 system parameters. The 39 system parameters, that characterize any engineering system, comprise the contradiction matrix. One parameter that is related to another rapidly degrades as the other value is increased. The relationship between the two factors produces the fundamental engineering paradox in the issue. Thus, the creative principles should be selected to direct the contradiction’s resolution by crossing the parameters in the contradiction matrix. The “then” and “but” statements of the engineering contradiction are then linked to the 39 system parameters of TRIZ based on their relevance and suitability as shown in Table 2.

To find the SP to resolve EC1, the “then” statement is paired with parameter 6 (P6: area of stationary object) because a single area in the classroom is occupied by many students. In the “but” statement, the “but” statement is paired with parameter 38 (P38: extent of automation), as the situation reflects the user’s limitation in switching the units on and off manually. The improving parameter (P6) and the deteriorating parameter (P38) are intersected within the TRIZ matrix of contradictions. This contradiction matrix comprises combinations of inventive principles from the 40 inventive principles of TRIZ. This list is a set of principles that help in generating creative solutions [20,21]. The result of the intersection is a TRIZ inventive principle (IV) known as the feedback principle (IV #23: Feedback). This principle states that a designer should introduce feedback into their system to improve a process or action. Alternatively, if feedback is already used, the designer should change its extent or influence.

On the other hand, to find the SP to resolve EC2, the “then” statement is paired with parameter 33 (P33: ease of operation) because the desired system should be expected to be easily operated and modifiable from the existing system implemented to keep the cost low and affordable. In the “but” statement, the “but” statement is paired with parameter 15 (P15: duration of action of moving object), as the situation reflects the need for the user to have constant movement to trigger the lighting continuously in conventional PIR sensor system implementation. The improving parameter (P33) and the deteriorating parameter (P15) is intersected within the TRIZ matrix of contradictions, giving a few recommended TRIZ inventive principles. From the options, the TRIZ inventive principle (IV) known as the self-service principle (IV #25: Self-service) is selected. This principle states that a designer should introduce auxiliary helpful functions into an object to serve its purpose and organize itself. Table 3 shows the summary of the inventive principles identified from the intersection of the selected TRIZ system parameters (SPs) in the contradiction matrix. In the end, the feedback principle (IV #23) was selected to solve EC1, while the principle of self-service (IV #25) was selected to solve EC2.

Hence, to address the feedback principle (IV #23) to solve the problem of the user’s limitation in manually turning their equipment on and off, an automatic mechanism could be introduced based on a specific feedback or trigger system. The idea struck when considering that trigger systems are commonly used in security alarms. Some of the alarm systems are triggered by human movements [9,10]. By avoiding the common method of installing PIR sensors on the ceiling to detect human motion, we use the principle of feedback such as in alarms for human presence detection, by installing a dual PIR sensor in the entrance and exit way of the classroom to detect human movements when entering or leaving the classroom to develop a smart lighting and control system that enables automatic switching on, and off of lighting. Furthermore, to address the self-service principle (IV #25) to solve the problem of conventional PIR sensor’s dependency on constant human movement to enable the triggering of lighting, we look into auxiliary helpful functions to be introduced into the system to enable it to automatically detect the number of people in a classroom. The idea struck when considering a counter system that can count the number of people entering a classroom and the number of people exiting a classroom. By performing simple arithmetic of addition or subtraction based on the number of people entering and exiting the classroom, the system can sense the presence of people in the venue accurately. Hence, a trigger-counting capability is proposed to be implemented into the system. Combining the idea of dual PIR sensors installed in the entrance and exit way of the classroom and the idea of a counter system, the concept of designing a smart lighting and control system for the classroom based on counter application with dual PIR sensors is generated.

### 3.2. Proposed Prototype

**Figure 2:**
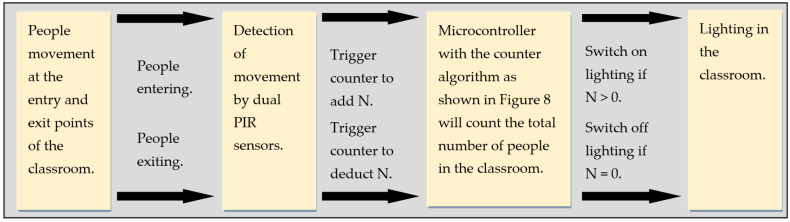
Context diagram of the system.

The smart lighting and control system proposed allows lighting and utilities to be switched on and off automatically based on human movement when entering or leaving a classroom. It is also able to count the total number of people (represented as N) in the classroom. The whole idea of the operation is explained in the context diagram of the system in Figure 2. Human movement in the environment which is the entrance and exit door of a classroom will be an acting stimulus upon the dual PIR sensors installed. Human movement can be categorized as entering the classroom and outgoing or exiting the classroom. Based on the number of times the sensors are triggered, a signal will be sent to the counter to count the number of people entering or leaving a classroom. Based on the number of people counted by the counter, the decision will be made by the microcontroller to request to switch on or off the electrical appliances connected to the system output which is the lighting in the classroom. The scenario cover can be when no one is in the classroom, when the first person enters the classroom, when many people enter or leave the classroom and when the last person leaves the classroom which is further elaborated below.

**Figure 3:**
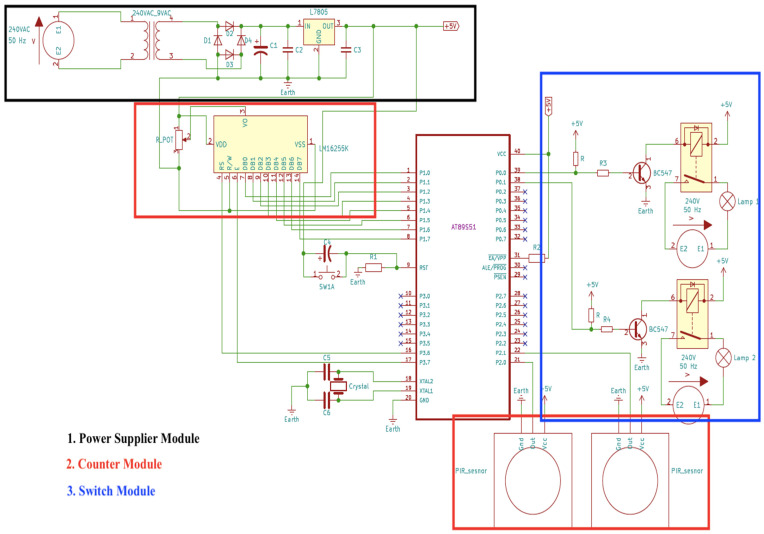
Schematic diagram of the proposed system.

Initially, when no one is detected in a vacant classroom, the counter is set to zero. When a person enters the vacant classroom through a door equipped with the dual PIR sensor, it detects movement and triggers the counter module to start counting to 1 and activate the electrical devices in the room. Even if several people enter the classroom, the output loads remain in switch-on mode as long as the total number of persons detected in the classroom is more than or equal to 1. On the other hand, when people leave the class through the door, the dual PIR sensor will also detect them exiting and then trigger the counter to subtract the number of people leaving from the total number of people in the classroom. As soon as the last person leaves the classroom, the counter shall count back to zero and the system shall automatically switch off the electrical appliances. If the system detects no person left in the classroom, it shall remain switched off in all connected electrical appliances. The proposed system is shown schematically in Figure 3.

It consists of three modules, namely the power supply module, the counter module, and the switching module which are all connected and controlled by a single AT89S51 microcontroller. Developed by Microchip Technology Inc. in the USA, the AT89S51 was selected due to its low-power requirement and high-performance CMOS 8-bit microcontroller with 4KB of ISP flash memory [32]. Referring to Figure 2, the power supply module (highlighted in the black box) supplies and converts the AC source to the DC source required for the operation of this system. The counter module (highlighted in the red box), which is attached to the entrance door of a venue, counts the number of people entering and leaving a room. The third module is the switch module (highlighted in the blue box), which is connected to the AC source and automatically switches on the output electrical load that is connected, based on the count data provided by the counter module. Each of the modules shall be further elaborated in more detail below.

#### 3.2.1. Power Supply Module

**Figure 4:**
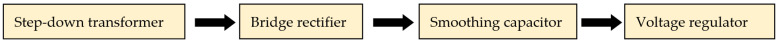
Power supply module.

The power supply module contains a step-down transformer, a bridge rectifier, a smoothing capacitor, and a voltage regulation module as portrayed in a block diagram in Figure 4. The step-down transformer converts the high AC input voltage of 240 V, 50 Hz into a lower AC output voltage of 9 V, 50 Hz. The full wave rectifier circuit converts the AC output voltage to the DC voltage. It uses two junction diodes (D1 with D4 and D2 with D3) to convert both half-cycles of the AC voltage waveform into a series of voltage pulses as shown in Figure 3. Then, the voltage pulse passes through a smoothing capacitor, C1, to lessen any ripple voltage that exists even further [33,34,35]. Finally, the voltage regulator L7805 module regulates and distributes the required voltage at 5 V in DC to power the microcontroller, the counter module, and the switch module.

#### 3.2.2. Counter Module

**Figure 5:**
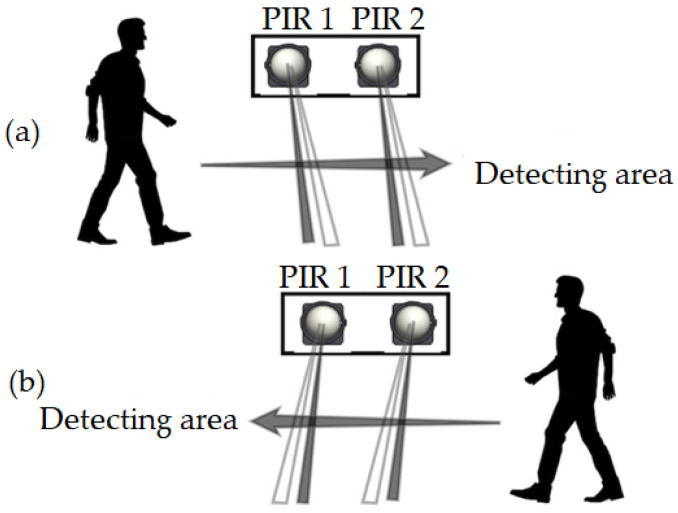
The sequence of movement of a person between the dual PIR sensors namely PIR 1 and PIR 2 installed at the door of a classroom, (**a**) showing the detection when a person enters a classroom and (**b**) showing the detection of a person exiting the classroom.

A PIR sensor detects human motion by sensing changes in the infrared radiation emitted by warm-bodied objects, such as humans, within its field of view. They are preferred because PIR sensors are easily available, are less costly, and have good energy efficiency [7]. In addition, the PIR sensor is not affected by sunlight or other visible light, making it suitable for indoor use. The PIR sensor contains a pyroelectric sensor that converts the incident infrared flux into an electrical output in two steps: the absorption layer converts the radiant flux into a temperature change and the pyroelectric element converts the thermal flux into electrical energy. Two PIR sensors used in this system are installed side by side on the door of a classroom. The output signal generated by the PIR sensor upon detection of human motion is sent to the microcontroller to decide if a person is entering or exiting a classroom. When a movement is detected by the PIR sensors, it generates a high output on the output pin. Referring to Figure 5, the sequence of activation of the sensors between PIR 1 and PIR 2 or vice versa will determine if a person is entering or exiting the classroom. If detection is triggered from PIR 1 first and later to PIR 2 (left to right), this distinguishes the movement of a person entering a classroom. On the other hand, if the sequence of activation of the sensors is from PIR 2 first and later to PIR 1 (right to left), this distinguishes the movement when a person leaves the classroom.

**Figure 6:**
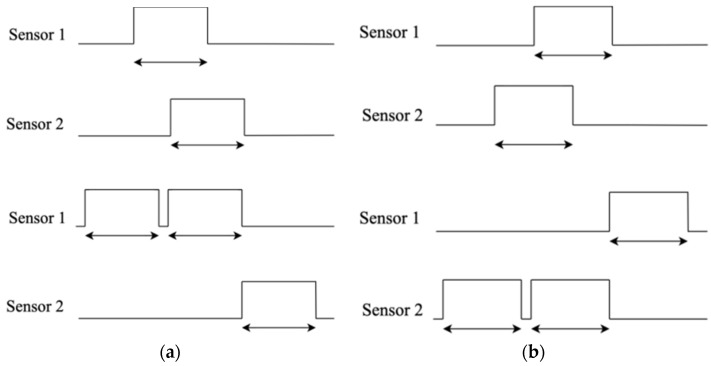
Signal timing diagram. (**a**) Signal from PIR sensors during a person entering a classroom; (**b**) signal from PIR sensors during a person exiting the classroom.

Therefore, the sensors produce an output that can be seen in Figure 6. This distinguishes entering or leaving a classroom by analyzing the time at which the sensors were triggered. The signals are fed into the AT89S51 microcontroller to be compared to determine whether a person enters the classroom or not and to trigger the counter to add or deduct the number of people in the room. The system only detects that someone is entering the classroom if both sensors are triggered accordingly. If someone stops in front of the first sensor, the system shall wait for the person to pass through the second sensor to trigger counting. However, if someone passes the first sensor and instead of passing the second sensor, turns around to leave the same way they came, the microcontroller resets the entry count of the first sensor after two seconds. This method also works for exit detection. The AT89S51 microcontroller works in real-time and simultaneously acts as a counter to count the number of people entering or leaving the classroom. The total number of people in the classroom is then displayed on a 16 × 2 LCD screen of an LM16255K module used in this system.

#### 3.2.3. Switch Module

**Figure 7:**
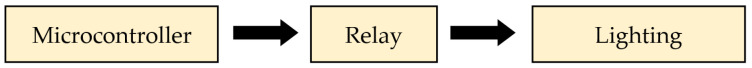
The block diagram of the switch module.

The switch module consists of the AT89S51 microcontroller and two units of relay to control the two outputs, which can be connected to AC output loads such as lighting to an external power source as shown in the block diagram in Figure 7. The signal received from the dual PIR sensors activation will activate the counting in the microcontroller which will later send an output signal to the relay to connect (switch on) or disconnect (switch off) the lighting from the external power source. The relays only act as electrically operated switches that open and close the circuits by receiving electrical signals from the microcontroller. Appropriate relays should be selected based on their power limit correlated to the external power supply power rating and output loads power rating to which they are connected. The relay modules used in this circuit are actively low and turn on when they receive a low-state output from the microcontroller. If the total number of people in the room is greater than zero, the switch module switches on the output loads. If, on the other hand, the room is empty and no one is present, the electrical loads are switched off by the relay. No human interface is required as the process is automatically controlled by the system.

#### 3.2.4. Operation Flowchart for the Algorithm

**Figure 8:**
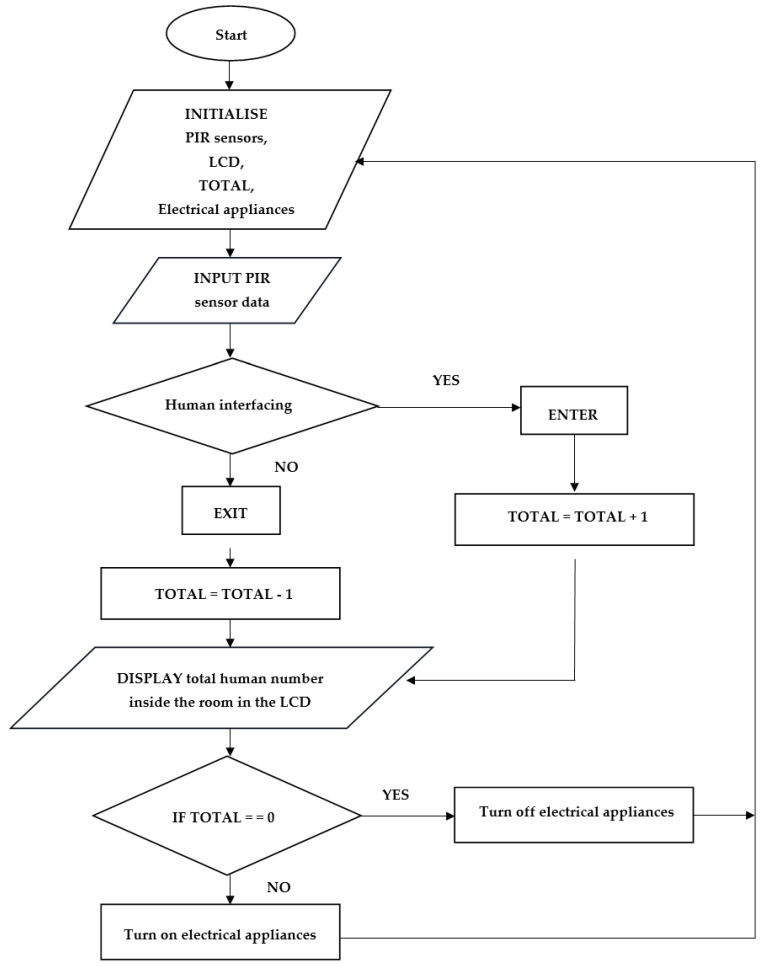
Flowchart of the smart electricity triggering system.

The operational process is an endless loop that begins when the PIR sensors detect a person. When someone enters the classroom, 1 is added to the total, which is initially zero. When someone leaves the classroom, the total number is subtracted by 1. The total number is shown on the display in real-time. When the total number is zero, the electrical devices are switched off, and when the number is greater than zero, the electrical devices are automatically switched on. The flow chart of the system shown in Figure 8 elaborates the whole algorithm used in programming the AT89S51 microcontroller in correlating the dual PIR sensors sensing to the counter which calculates the total number of people in the classroom as well as the switching sequence of the output loads.

## 4. Result and Discussion

**Figure 9:**
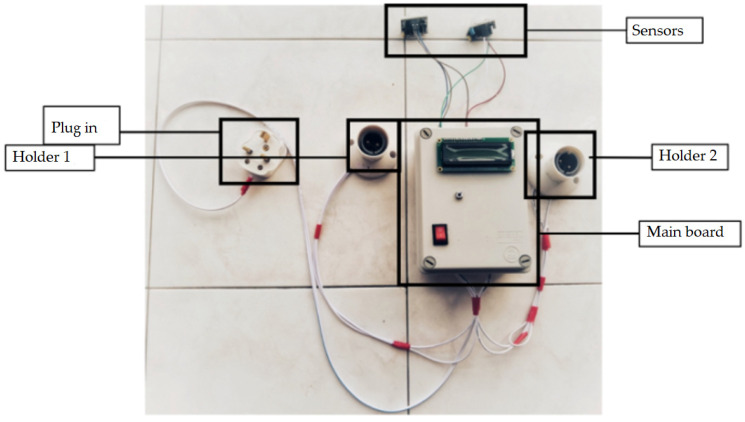
Outlook of the smart electricity triggering system.

The results and discussion section for the smart lighting and control system is divided into 3 sections, namely the discussion on the power supply module, the testing and validation of the functionality of the system, and the power consumption of the system. Figure 9 shows the outlook of the proposed system.

### 4.1. Power Supply Module Discussion

**Figure 10:**
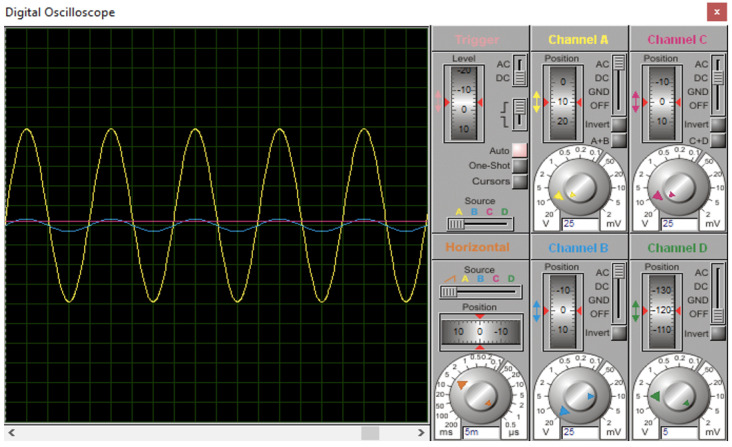
Waveform simulation for power supply module.

The power supply module converts the 240 V AC to 5 V DC. First, the step-down transformer converts the 250 V AC, 50 Hz input supply to 9 V AC with 200 mA. Then, the rectifier converts the 9 V AC into 9 V DC, which later runs through the voltage regulator and regulates the output voltage to 5 V DC. The power supply module is tested in the laboratory using a GDS-1072-UGW INSTEK digital oscilloscope from Good Will Instrument (GW Instek), Taiwan which displays the input and output voltage waveforms as shown in Figure 10. The yellow waveform is the input voltage of 240 V AC from the transformer, the blue waveform represents the output voltage of 9 V AC from the transformer and the pink waveform represents the output voltage of 5 V DC from the voltage regulator.

### 4.2. Testing and Validation of the System Functionality

The testing of the functionality and effectiveness of the system is explained in this section. At the initial stage, we start the testing condition with no one in the classroom, hence, the electrical output loads are switched off. Next, we simulate the condition of a person entering the classroom and the system can detect the person via the two PIR sensors which trigger the system to activate the electrical output loads. Then, a simultaneous simulated condition of people entering and leaving the classroom is carried out to check the effectiveness of the system in detecting the number of people in the classroom to correlate to the triggering of the output loads. As indicated in Table 3, the outputs are continuously switched on if there is a person in the classroom and switched off when the last person leaves the classroom with no one detected anymore. Table 4 depicts the result of the testing the prototype to summarize how the output loads (which are represented by two light bulbs) react to the testing conditions of people entering and exiting the classroom.

**Figure 11:**
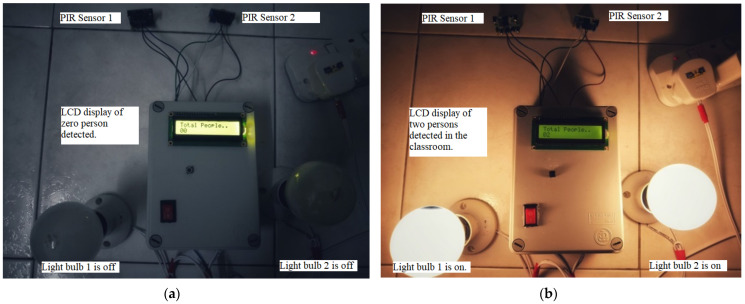
Output conditions when the system senses and counts the presence of people in a classroom and triggers the loads correspondingly. (**a**) Detection of non-presence of people in a classroom and counter shows zero with the loads switched off; (**b**) detection of the presence of people in the classroom with counters showing the detected number of persons and the loads switched on.

At the initial stage, the counter reading of the microcontroller is first set to zero and the output loads (light bulbs) are switched off, as shown in Figure 11a. When the system detects an entry into the room, it starts counting and shows the total count as one on the LCD. When another person enters the classroom, the system leaves the bulbs on and shows the total count as two on the display, as shown in Figure 11b. When one of the individuals leaves the classroom, the system detects this, subtracts one from the total count, and continues to keep the bulbs switched on. As long as one person is still in the classroom, the total number remains one. When the system detects that the last person is leaving the classroom, it subtracts the total number to show zero on the display and switches off all the output loads automatically. This shows the effectiveness and reliability of the system in detecting and counting the number of people in the classroom to correlate with the decision to switch on or off the electrical appliances accurately. Hence, the ability of the system to detect the movement of people entering and exiting the classroom and its ability to correlate with the need to switch on or off the electrical output loads accurately based on the setting criteria of the counter, signifies the success of the prototype in meeting its objectives and purposes. The detection range of the PIR sensors embedded at the entry and exit point of the classroom is tuned to be approximately 2.5 m.

### 4.3. Power Consumption of the System

An evaluation of the power consumption of the prototype is conducted. It measures the voltage in volts and current in amperes used to operate the system. To determine energy consumption, the power is determined by multiplying the voltage magnitude by the current. The power consumption of resistors, capacitors, crystals, and diodes is negligible as it is less than 1 µA. Table 5 summarizes the power consumption of the system in the on-state, which is cumulatively at 490.5 mW including all the major electronic components. The total power consumed to operate the prototype is relatively low; hence it shall be cost effective to operate the system by the user and in line with the energy saving initiative.

## 5. Limitations and Prospects

The smart lighting and control system operated with a dual PIR sensor, while effective in many scenarios, has certain limitations such as a limited sensing range to detect the motion of people entering and exiting the classroom. PIR sensors typically sense motion within a specific radius, and their effectiveness diminishes with distance. This limitation might result in areas where the sensor cannot detect movement if placed inappropriately at a door. Hence, the dual PIR sensors are limited to being placed at the top of the door vertically facing down to enable its range of sensing to be suitable for detecting people’s movement in and out of the classroom. Besides that, the line-of-sight requirement by the PIR sensor requires a clear line of sight to detect motion accurately. Obstacles or physical barriers between the sensor and the moving object can reduce the sensor’s effectiveness. This can result in situations where motion is not detected, or there are false positives due to interference. This limitation can occur if the dual PIR sensors are blocked. In addition, PIR sensors are designed for single-point detection and may struggle to cover large or irregularly shaped spaces of entrance or exit points effectively. In such cases, additional sensors may be required, increasing the complexity and cost of the system. Besides that, PIR sensors can have a slight delay in response time, especially when switching the lights on. This delay might be noticeable in situations where immediate lighting adjustments are crucial, potentially impacting user experience and safety. However, for simple adaptation in the classroom to switch on the lighting, it remains adequate for these needs. Furthermore, PIR sensors are more effective at detecting larger motions rather than subtle movements. This limitation can be a drawback in applications where precise motion detection is essential, such as in environments with minimal human movement. Lastly, PIR sensors can be sensitive to changes in temperature, leading to false triggers. For example, sudden temperature fluctuations, drafts, or the presence of animals may cause the sensor to activate the lighting system unnecessarily. Thus, to overcome these limitations, the future works for a comprehensive smart lighting system may incorporate a combination of sensor technologies, such as ultrasonic sensors, microwave sensors, or light sensors, to enhance overall performance and reliability.

## 6. Conclusions

In summary, this study introduces an innovative and efficient solution of an optimized and automated switching system based on human motion detection equipped with counters which is specifically designed for smart lighting and classroom control. The developed prototype not only detects the presence of individuals within a confined space but also accurately counts the number of occupants. By automatically activating the lighting when anyone is present and deactivating them when the last person exits the classroom, the system effectively addresses the issue of electricity wastage resulting from human negligence in turning off appliances. The implementation involves the use of dual PIR sensors with a counter system, offering a sophisticated yet user-friendly approach to managing multiple entries and exits.

This advancement resolves the shortcomings of traditional systems that rely on manual switching or continuous body movements to keep automated systems alert. The proposed solution not only streamlines the process but also contributes to a reduction in power consumption and associated electricity costs. The applicability of this system extends to spaces with a high volume of occupants, such as university lecture theatres, library spaces, assembly halls, offices, and others by providing an intelligent and automated approach to lighting control. In essence, this study not only pioneers a practical solution for energy conservation but also sets the stage for a more sustainable and user-friendly future in the management of electrical appliances in shared spaces.

## Figures and Tables

**Table 1:** Comparison table for existing approaches to the proposed method.

No.	Method Employed	Key Features	Significant Contributions	Limitations	Utilization of PIR Sensor	Dependence on Constant Movement of User for Operation	References
1	An autonomous lighting control system that detects the presence of humans and controls the intensity of the lamp.	Usage of a light sensor, a passive infrared (PIR), and an infrared (IR) sensor to detect the presence of human.	The system can measure and analyze the brightness of light according to the surrounding exposure and provides easy installation.	Depends on detection of continuous movement of people in the classroom for continuous control of light intensity.	Yes	Yes	[11]
2	Application of pyroelectric infrared sensors to be embedded into a non-terminal indoor localization system mounted on the ceiling to increase the precision in estimating the location of a target inside a room.	Usage of a PIR detection module (PDM) system made of two PIR modules to collect infrared signals for human sensing and estimation of the person’s location in a room.	The proposed system with 2 PIR modules achieves simplified system design and better estimation accuracy.	Depends on detection of continuous movement of people in the room for estimation of the target location.	Yes	Yes	[12]
3	Application of fuzzy logic algorithms to analyze video data to identify human presence.	Detect human presence in surveillance video by extracting the variety of human skin color with a fuzzy approach from the selected video frame.	The approach suggested improves the effectiveness of human detection in a video.	Depends on CCTV video frame data only which is not applicable for a conventional classroom without any CCTV installation.	No	No	[13]
4	Application of explicit skin color space thresholding and minor motion detection techniques to accurately detect human presence in visual data.	Implement skin color detection by using explicit thresholding of CbCr color channels and usage of morphological differences of multiple consecutive images for minor motion detection	The approach is efficient in detecting human presence based on skin color and motion in frame of video as well as identifying the location of the human within the frame.	Depends on CCTV video frame data only which is not applicable for a conventional classroom without any CCTV installation	No	No	[14]
5	Regulation of lighting based on the presence of humans using relay control and mobility.	Employs an infrared remote-control mechanism to switch on or off an energy system in the absence of humans	The research produces a low cost, portable microcontroller-based automated room light controller applicable for energy waste management.	The prototype experiences a limited range for sensing objects/people.	No	No	[15]
6	Application of an Arduino-based automatic lighting and control system connected to relay control to detect human presence with coupling to Android mobile app and Bluetooth control to enable control of the lighting with voice command.	The research proposed the division of the classroom into grids to control lighting in particular areas of the classroom based on the presence of human.	An automatic lighting control system is developed which can detect human location in its grid for switching appliances on or off.	The prototype with additional features proposed can incur a higher cost of fabrication.	No	No	[4]
7	Application of counter system with dual PIR sensors to enable smart lighting and control system for classroom.	Usage of two PIR sensors and counter to automatically count the amount of people in the classroom without dependence on continuous human motion detection.	Explained in 1.1 Research Contribution.	Explained in 5. Limitations and Prospects.	Yes	No	Current proposed method

**Table 2:** List of 39 System Parameters of TRIZ.

No.	System Parameter	No.	System Parameter
1	Weight of Moving Object	21	Power
2	Weight of Stationary Object	22	Loss of Energy
3	Length of Moving Object	23	Loss of Substance
4	Length of Stationary Object	24	Loss of Information
5	Area of Moving Object	25	Loss of Time
6	Area of Stationary Object	26	Quantity of Substance
7	The Volume of the Moving Object	27	Reliability
8	The Volume of the Stationary Object	28	Measurement Accuracy
9	Speed	29	Manufacturing Precision
10	Force	30	Object Affected Harmful Factors
11	Pressure or Stress	31	Object-Generated Harmful Factors
12	Shape	32	Manufacturability
13	Stability of the Object’s Composition	33	Ease of Operation
14	Strength	34	Repairability
15	Duration of Action of Moving Object	35	Adaptability or Versatility
16	Duration of Action of Stationary Object	36	Device Complexity
17	Temperature	37	Difficulty in Detecting and Measuring
18	Illumination Intensity	38	Extent of Automation
19	Use of Energy by Moving Object	39	Productivity
20	Use of Energy by Stationary Object		

**Table 3:** Summary of inventive principles identified from the intersection of system parameters.

Details	EC1	EC2
“then” part (with system parameter)	then such electricity wastage caused by the students who do not switch off the lighting before exiting the classroom should be avoided (SP #6 Area of stationary object)	then such a system must be modified from the existing system to avoid huge changes incurring additional costs (SP #33 Ease of operation)
“but” part (with system parameter, SP)	but this required an intelligent autonomous system to control the switching of the lighting based on human presence detection to avoid dependency on human manual control (SP #38 Extent of Automation)	but the current cheap system is mostly designed with conventional PIR sensors which depend on constant human motion to enable continuous triggering of lighting (SP #15 Duration of action of moving object)
Inventive principle (IV)	IV #23: Feedback	IV #29: Pneumatics and Hydraulics IV #3: Local quality IV #8: Anti-weight IV #25: Self-service
Selected	IV #23: Feedback	IV #25: Self-service

**Table 4:** Results based on number of people detected in a room.

Condition	Total Number	Effect
Initial	00	2 Light Bulbs are OFF
Entrance	01	ON two light bulbs
Entrance	02	ON two light bulbs
Exit	01	ON two light bulbs
Exit	00	OFF two light bulbs

**Table 5:** Power consumption by the system.

Components	Voltage (V)	Current (mA)	Power (mW)
AT89S51 Microcontroller	5	25	50
2 PIR Sensors	5	0.1	0.5
2 Channel relay driver	5	72	360
LCD	5	16	80
			**Total power:** 490.5

## Data Availability

Data are contained within the article.
